# Adaptive Evolution Signatures in *Prochlorococcus*: Open Reading Frame (ORF)eome Resources and Insights from Comparative Genomics

**DOI:** 10.3390/microorganisms12081720

**Published:** 2024-08-20

**Authors:** Sarah Daakour, David R. Nelson, Weiqi Fu, Ashish Jaiswal, Bushra Dohai, Amnah Salem Alzahmi, Joseph Koussa, Xiaoluo Huang, Yue Shen, Jean-Claude Twizere, Kourosh Salehi-Ashtiani

**Affiliations:** 1 Center for Genomics and Systems Biology (CGSB), New York University-Abu Dhabi, Abu Dhabi P.O. Box 129188, United Arab Emirates; sd145@nyu.edu (S.D.); drn2@nyu.edu (D.R.N.); weiqifu@zju.edu.cn (W.F.); akj4@nyu.edu (A.J.); dohaibushra@gmail.com (B.D.); amnah.alzahmi@nyu.edu (A.S.A.); jk150@nyu.edu (J.K.); jean-claude.twizere@uliege.be (J.-C.T.); 2 Division of Science and Math, New York University-Abu Dhabi, Abu Dhabi P.O. Box 129188, United Arab Emirates; 3 Department of Marine Science, Ocean College, Zhejiang University, Zhoushan 316021, China; 4 Helmholtz Center Munich, Institute of Network Biology (INET), German Research Center for Environmental Health, 85764 Munich, Germany; 5 Laboratory of Viral Interactomes Networks, Unit of Molecular & Computational Biology, Interdisciplinary Cluster for Applied Genoproteomics (GIGA Institute), University of Liège, 4000 Liège, Belgium; 6 Department of Biology, New York University, New York, NY 10012, USA; 7 Department of Chemical and Biological Sciences, Montgomery College, Germantown, MD 20850, USA; 8 Genome Synthesis and Editing Platform, China National GeneBank (CNGB), BGI-Research, Shenzhen 518120, China; huangxl@siat.ac.cn (X.H.); shenyue@cngb.org (Y.S.); 9 Shenzhen Institute of Advanced Technology, Chinese Academy of Sciences, Beijing 100045, China

**Keywords:** *Prochlorococcus*, comparative genomics, light adaptations, deep learning, endogenous viral elements, MED4, NALT1A

## Abstract

*Prochlorococcus*, a cyanobacteria genus of the smallest and most abundant oceanic phototrophs, encompasses ecotype strains adapted to high-light (HL) and low-light (LL) niches. To elucidate the adaptive evolution of this genus, we analyzed 40 *Prochlorococcus marinus* ORFeomes, including two cornerstone strains, MED4 and NATL1A. Employing deep learning with robust statistical methods, we detected new protein family distributions in the strains and identified key genes differentiating the HL and LL strains. The HL strains harbor genes (ABC-2 transporters) related to stress resistance, such as DNA repair and RNA processing, while the LL strains exhibit unique chlorophyll adaptations (ion transport proteins, HEAT repeats). Additionally, we report the finding of variable, depth-dependent endogenous viral elements in the 40 strains. To generate biological resources to experimentally study the HL and LL adaptations, we constructed the ORFeomes of two representative strains, MED4 and NATL1A synthetically, covering 99% of the annotated protein-coding sequences of the two species, totaling 3976 cloned, sequence-verified open reading frames (ORFs). These comparative genomic analyses, paired with MED4 and NATL1A ORFeomes, will facilitate future genotype-to-phenotype mappings and the systems biology exploration of *Prochlorococcus* ecology.

## 1. Introduction

Cyanobacteria are the major primary producers in aquatic environments [1,2]. They are model organisms for studying photosynthesis, carbon and nitrogen assimilation, evolution, adaptation to environmental conditions, and specialized biotechnology applications [3]. Among cyanobacteria, *Prochlorococcus* and *Synechococcus* are the dominant primary producers in marine ecosystems and are responsible for the fixation of massive quantities of atmospheric carbon [4,5].

The species belonging to *Prochlorococcus* are a genus adapted to thrive in oceanic conditions of high oxygen and low nutrient levels [6]. The members of this genus are adapted to radiation and can grow at a range of depths in the ocean. There is a primary distinction between the two major classifications for high-light (HL)- and low-light (LL)-adapted strains [7]. Gene gain and loss frequently occur in *P. marinus* [8]. Based on comparing the genomes of 12 isolated strains of *P. marinus*, a set of 1273 shared genes was identified as a core, conserved gene set. These genes underlie the essential processes for *Prochlorococcus marinus* in any environment, while the remaining genes probably play roles in niche adaptation [9]. Fifty different *P. marinus* strains have been isolated and sequenced [10], with the strains MED4 [11], SS120 [12], and MIT9313 [13] being the first to be sequenced in 2003. The current genome annotations for *P. marinus* offer a detailed description of the genes, proteins, sequences, and functions, providing a base dataset for bioinformatic analyses; however, most predicted gene products from the currently available genome annotations remain experimentally uncharacterized. Thus, a lack of resources limits the experimental explorations of genotype-to-phenotype associations.

In this paper, the comparative analyses of *P. marinus* genomes and their protein family (Pfam) domains are described, providing interpretations of their adaptation to HL and LL conditions. Our approach involves dimensional reductions to detect clusters of differential gene content among the strains and the use of robust statistical methods and artificial neural networks to identify sets of key Pfams that can distinguish the features of HL and LL strains. Using this approach to explore *P. marinus* adaptation can provide insights into aquatic ecology, carbon, and climate impacts. Furthermore, we report on the construction of nearly complete MED4 and NATL1A ORFeomes through de novo chemical DNA synthesis, generating the first set of available cyanobacteria ORFeome resources. The synthesized ORFs were sequence-verified, and the selected ORFs were tested using recombinational cloning, thereby forming expression vectors available for a systematic experimental assessment. Together, the computational and biological resources generated in this work will substantially advance cyanobacterial and variable depth studies and provide support for the multifaceted ecological and evolutionary fields of research using cyanobacteria as model organisms.

## 2. Material and Methods

### 2.1. Protein Family Domain Prediction

The *P. marinus* genomes were downloaded from the NCBI/Genome assembly and an annotation report for 42 available isolates. The gene annotation yielded a set of peptide predictions serving as a base for HMM alignment with the Pfam-A-v31.1 database. The command was ‘*hmmsearch–noali-E 0.000000001–cpu 28–domtblout $OUT $IN.aa.fa*’. The HMMer version 3.4 (http://hmmer.org, accessed on 1 March 2023) user guide (http://eddylab.org/software/hmmer/Userguide.pdf, accessed on 1 March 2023) provides in-depth descriptions of the reported values, including sequence coordinates for alignment and matches with HMM models, E-values, percent identity, and bias.

The predicted proteins were also analyzed for similarity to known proteins using BLASTP (v2.2.31).

### 2.2. Hierarchical Bi-Clustering

The assemblies for *P. marinus* used in this paper predicted 1196 Pfams that were used for the bi-clustered heatmap visualization in Morpheus [14]: a web tool for visualizing the clustering of multivariate data (https://software.broadinstitute.org/morpheus/, accessed on 1 September 2023). Clustering was performed on Pfams and species (bi-clustering) using Pearson correlation scores.

The complete sets of the resulting annotations are available as transcripts and proteins in fasta format (.fa) in the Supplementary File, Data S1.

### 2.3. Response Screening

The parsed HMMsearch results (i.e., the Pfam matrices) were used in comparative analyses. In brief, normalized sum bit scores for Pfams in the 40 surveyed *P. marinus* strains (i.e., ‘input matrix’; see Data S2) were used as input for the false-discovery rate (FDR)- and outlier-corrected batch *t*-tests (response screens). The means were analyzed using analysis of variance (ANOVA) tests, where each comparison’s *p*-value, logworth, false discovery rate (FDR, or adjusted) *p*-value, and FDR logworth were reported. The protein family averages between the HL and LL groups were compared and screened for significant differences and equivalencies (Table S3).

The standardized residues were used to compare means in >100,000 *t*-tests while controlling for FDR. Using robust Huber M-estimation (employing maximum-likelihood type estimators), the sensitivity of the tests was reduced to outliers. In brief, the influence of outliers is reduced with this method through the minimization of their weights in the comparative statistics algorithm. The most useful information from these statistical tests for the general reader is provided in the ‘Response Screen Compare Means’ table, which includes the tests for all possible comparisons and results for practical equivalences and differences. The comparisons not passing either of these tests are labeled as ‘inconclusive’. The practical difference is the difference in means that are considered to be of practical interest. The standard deviation estimates were computed from their interquartile ranges (IQRs), where the estimate was σ = (IQR)/(1.3489795). The practical difference was computed as 6(σ) multiplied by 0.10 as a proxy for the practical difference proportion. The practical difference *p* values are provided for tests assessing whether the absolute value of the mean difference in Y between comparison levels is less than or equal to the practical difference. Low *p* values indicate that the absolute difference exceeds the practical difference, indicating that the difference in the comparison is of practical significance.

Practical equivalence *p* values were computed using the two one-sided tests (TOSTs) method to test for practical differences between means. The practical difference specifies a threshold difference for which smaller differences are considered practically equivalent. One-sided *t*-tests are constructed for two null hypotheses: the true difference exceeds the practical difference; the true difference is less than the negative of the practical difference. If both tests are rejected, this indicates that the absolute difference in the means falls within the practical difference. Therefore, the groups are considered practically equivalent. The practical equivalence *p* value is the largest *p* value obtained on the one-sided *t*-tests. The low practical equivalence *p*-values indicate that the mean response for the top comparison level is equivalent, in a practical sense, to the mean for the lower level.

### 2.4. Deep Learning ANN Analysis

We compared HL (*n* = 25) and LL (*n* = 15) *Prochlorococcus* strains to determine the main genetic differences that facilitate adaptation to HL and LL conditions. We used the top 20 significantly differing Pfams from the aforementioned response screen in the two groups (FDR *p* < 0.05) to analyze their differential genomic contents and provide a training set for an artificial neural network (ANN) model. Then, we constructed an ANN model using the top 20 significantly differing Pfams to distinguish between the HL and LL groups based on their genomic contents. The combined approach aimed to establish statistical integrity for batch comparisons while generating nonintuitive conclusions using the ANNs.

We compared the HL (*n* = 25) and LL (*n* = 15) *Prochlorococcus* strains to determine the main genetic differences facilitating adaptation to HL and LL conditions. We determined that there were significantly differing Pfams in the two groups (FDR *p* < 0.05), with the aim of analyzing their differential genomic contents and providing a training set for an artificial neural network (ANN) model. The predictive modeling using neural networks was carried out in the JMP Neural Networks module. Four sequential models were used, with three TanH nodes each and a learning rate of 0.1. In fitting, the covariates were transformed, and a squared penalty method was used. The Python code for the formulae necessary to reproduce the dNN is provided in Data S3. Both the training and validation runs yielded R2 values > 0.99 and misclassification rates of zero, indicating that the genomic contents of the HL and LL strains are sufficient to classify them into their respective environments.

### 2.5. VFam Analysis

Viral family sequences within genomes were discovered using HMMs built from Markov clustering and multiple sequence alignments [15] from viral proteins in RefSeq as of 2014. These VFams can detect viral sequences with high accuracy and did not cluster non-viral sequences into the Markov clusters in the published test sets. We used a strict 1 × 10^−9^ E-value to call VFam domains in the translated CDS (tCDS) from the *P. marinus* genomes. The command was ‘*hmmsearch—noali-E 0.000000001—cpu 28—domtblout $OUT $IN.aa.fa VFam.hmm*’.

The assemblies for *P. marinus* used in the manuscript predicted 3762 VFams that were utilized for bi-clustered heatmap visualization in Morpheus: a web tool for visualizing the clustering of multivariate data (https://software.broadinstitute.org/morpheus/, accessed on 1 November 2023). The clustering was performed on VFams and species (bi-clustering) using Pearson correlation scores, and heatmaps were visualized in Morpheus using sum HMM match scores as a color Z-depth, as in the Pfam analyses.

### 2.6. Metabolic Pathway Analysis

Interactive Pathways Explorer 3 (iPath3: https://pathways.embl.de, accessed on 15 January 2024) is a web-based tool for the visualization, analysis, and customization of various pathway maps [16]. iPath3 provides extensive map customization and data mapping capabilities. We used EC numbers (enzyme codes) from the BLAST2GO analysis for both MED4 and NATL1A and mapped the metabolic pathways, comparing unique and shared pathways between the two respective HL and LL strains.

### 2.7. Functional Annotation Analysis Using Blast2GO

The cDNA sequences of *P. marinus* MED4 and NATL1A were obtained from the National Center for Biotechnology Information (NCBI) and the Joint Genome Institute (JGI), respectively. The Blast2GO analysis was performed using Blast2GO Command Line Version 1.5.1 with the following parameters: (1) NCBI NR Database: a custom database was created using the latest NCBI non-redundant (nr) fasta file as of 1 October 2022, totaling 136 GB; (2) BLASTX Alignment: primary sequence alignments were obtained using BLASTX 2.13.0+ with the following parameters: -evalue 0.001, -show_gis, -max_target_seqs 5, -num_threads 60, -mt_mode 0, and -outfmt 5; (3) GO Database: the Gene Ontology (GO) database used was go_latest.obo as of October 2022. Blast2GO (B2G) is a BLAST-based tool used for the large-scale functional annotation of novel sequence data of non-model species. The focus of B2G development is the creation of a comprehensive, user-friendly, and research-oriented framework for large-scale function assignments [17].

### 2.8. ORFeome Synthesis and Cloning

The information about gene annotation and cDNA sequences were extracted for the complete genomes of MED4 and NATL1A strains from the EnsemblBacteria database (GCA_000011465.1 and GCA_000011485.1, respectively). The ORFs were synthesized in collaboration with Twist Bioscience (San Francisco, CA, USA; for the majority of the ORFeomes) and BGI (Shenzhen, China; 99 ORFs for the MED4 strain ranging from 78 bp to 4500 bp, including the longest gene sequences from 3000 bp to 4566 bp).

The synthesized ORFs are flanked by the following Gateway L1 and L2 sites:

ATTL1:caaataatgattttattttgactgatagtgacctgttcgttgcaacacattgatgagcaatgcttttttataatgccaactttgtacaaaaaagcaggctac

ATTL2:ttggacccagctttcttgtacaaagttggcattataagaaagcattgcttatcaatttgttgcaacgaacaggtcactatcagtcaaaataaaatcattatttg

DNA vectors were received in 96-well or 384-well plates as DNA and glycerol stocks. Uni9 primers can be used to amplify the ORFs (outside the ATTL sequences).

Uni 9 for (5′–3′): GAAGTGCCATTCCGCCTGACCT

Uni 9 rev (5′–3′): CACTGAGCCTCCACCTAGCCT

Gateway transfer and sequence validation

A set of MED4 ORFs was recombinationally cloned into yeast expression vectors (pAD and pDB) [18] using LR clonase (Invitrogen), according to the manufacturer’s instructions. The expression clones were subsequently transformed into chemically competent *E. coli* DH5α (Mix & Go competent cells Zymo 5z). The transformants were cultured as minipools in liquid LB with ampicillin (100 mg/L). Following growth in liquid media, the transformed bacteria were used as a source of the template for DNA extraction, with a GeneJET plasmid miniprep kit from Thermo Scientific (Waltham, MA, USA), following the manufacturer’s instructions. The cloning was verified with PCR using KOD hot-start DNA polymerase (Sigma-Aldrich, Merck KGaA, Darmstadt, Germany), with pAD and pDB-specific primers, and followed by gel electrophoresis.

A set of amplified clones was verified via sequencing, bi-directionally, at first base sequencing (Apical Scientific Sdn Bhd, Selangor, Malaysia). The forward and reverse sequences were mapped to the reference using ChromasPro version 2.1.5 (http://www.technelysium.com.au/ChromasPro.html, accessed on 1 October 2019).

AD-forward: 5′-CGCGTTTGGAATCACTACAGGG-3′

DB-forward: 5′-GGCTTCAGTGGAGACTGATATGCCTC-3′

Term-reverse: 5′-GGAGACTTGACCAAACCTCTGGCG-3′

## 3. Results

### 3.1. Decoding High-Light and Low-Light Associated Gene Sets from P. marinus Genomes

The *Prochlorococcus* species inhabiting different ocean depths deal with drastically different temperatures and availabilities of light and nutrients [6]. Additionally, their microbial cohorts, including grazers, viruses, and other planktonic species, differ significantly with depth [19]. Understanding the genomic basis for the depth and light adaption in *Prochlorococcus* can provide valuable insights into plant stress tolerance, marine ecology, and global biogeochemistry. The genomic differences between strains inhabiting different depths were hypothesized as potentially including variations in genes for photosynthesis, nutrient acquisition, and abiotic and biotic stress responses, which could reflect their adaptation to specific environmental conditions [20].

**Figure 1:**
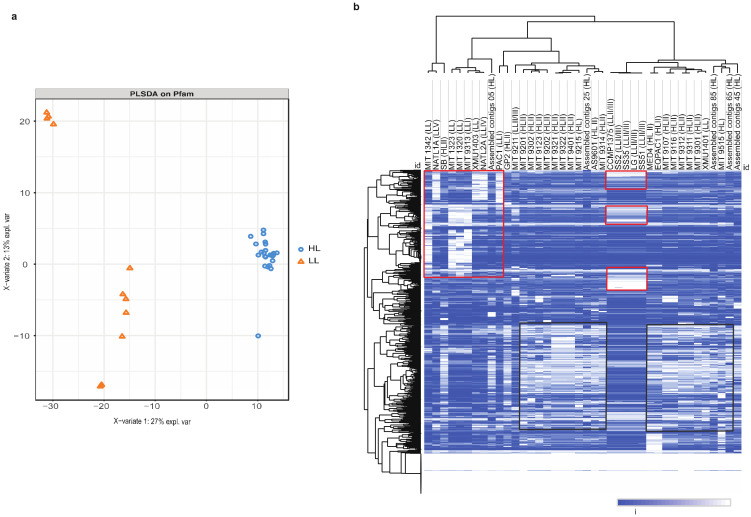
The partial least squares and hierarchical bi-clustering of protein family (Pfam) domains from 40 *P. marinus* isolates. (**a**) Cluster analysis using cluster-based partial least squares (PLS) of the 40 *P. marinus* strains. This method shows separate clustering for high-light (HL) and low-light (LL) strains represented in blue and orange, respectively; (**b**) heatmap of Pfam domains for 40 *P. marinus* species showing the clustering of distinct Pfam groups in HL and LL strains on the right and left sides, respectively. The 40 strains are represented on the X-axis with LL and HL; approximately 1000 different Pfams are represented on the Y-axis. The red and black rectangles highlight the different patterns of Pfam counts among clusters of LL and HL strains, respectively.

We generated Pfam domain annotations for 40 sequenced *Prochlorococcus* genomes (Supplementary Data S1). In total, 1196 Pfam domains were predicted from the encoded *P. marinus* proteins, and their normalized abundance was calculated from sum bit scores for each Pfam among the species (Table S1). In dimensionality reduction analyses, the HL and LL strains showed distinct Pfam clustering. The clustering from the partial least squares (PLS) method is shown in Figure 1a. Hierarchical bi-clustering using Euclidean distances also revealed distinct clustering patterns (Figure 1b). Both analyses indicate that the environmental adaptation of these species can be recapitulated through Pfam differential retention and copy number variation. The key genes containing these Pfams were extracted and analyzed for their differential representation among the strains. We observed an average ratio of ~5-fold higher for the LL strains compared to the HL strains for a GHMP kinase domain (inc. homoserine kinases, galactokinases, and mevalonate kinases (PF00288)). The N-terminal domains of the GHMP kinase family and the photosystem II (PSII) protein domains were highly represented in the LL strains. PSII critically increases the efficiency of photosynthetic light capture, maximizing energy production in low-light conditions. In addition, protein kinases are essential in the process of phosphorylation and photosynthetic acclimation in the core of PSII [21].

We observed a ferrous iron transport protein B at a ~2-fold higher copy number compared to the HL strains. This Pfam is involved in the electron transport chain processes (e.g., photosynthesis and respiration) [22]. In low-iron conditions, cyanobacteria acclimate through the modification of electron transport and metabolic pathways and by managing iron levels through specific uptake, transport, and storage mechanisms [23]. In *Synechocystis* sp. PCC 6803, the FitB protein, a ferrous iron transporter, is key in internal iron transport, becoming more active under conditions of iron scarcity to aid in this adaptation [24]. In the HL strains, PF16881—the N-terminal domain of lipoyl synthase of the radical_SAM family—was found in ~10-fold higher copy numbers compared to LL strains (Table S1). Lipoyl synthase enzymes are responsible for catalyzing the final step in the biosynthesis of the lipoyl cofactor. This process involves the incorporation of two sulfur atoms at the C-6 and C-8 positions of the octanoyl moiety attached to the lipoyl domains of proteins [25]. These enzymes play crucial roles in the biosynthesis of lipoic acid as co-factors for several enzyme complexes involved in central metabolism. Lipoic acid is essential for the proper functioning of various metabolic pathways demonstrated in bacteria (*Escherichia coli* and *Bacillus subtilis*) [26]. Although the specific role of lipoyl synthase in HL-adapted cyanobacteria has not yet been defined, its function suggests that higher copy numbers represent a metabolic adaptation to HL conditions.

We found that an ABC-2 transporter (PF06182) was present in HL strains at >3-fold copy numbers compared to LL strains (*p* = 1.59 × 10^−24^; Tables S1 and S4). In *Prochlorococcus*, the ABC transporters promote cellular viability in HL upper photic zone conditions by facilitating the transport of essential nutrients, antioxidants, and other molecules [11]. In the upper photic zone, nutrient concentrations (such as nitrogen, phosphorus, and iron) can be limiting for growth [27]. The ABC transporters can facilitate sparse nutrient uptake by actively transporting them into the cell against a concentration gradient. Efficient nutrient acquisition can give *Prochlorococcus* a competitive advantage in these nutrient-poor environments. These HL-specific ABC transporter domains may also be involved in the transport of antioxidants and other protective compounds, helping to maintain the cellular redox balance and protecting against oxidative stress [28].

To gain an insight into the functional characteristics and biological processes associated with annotated *P. marinus* proteins, domain-centric gene ontology (GO) enrichment was performed. Here, Pfam input sets are used to calculate the enrichment for their associated GO-terms. This approach identified protein families and GO terms that were significantly over-represented in HL and LL strains. Some of the unique molecular functions enriched under LL conditions may be crucial for energy generation in such environments. These include oxidoreductase activity (acting on CH-OH group of donors) (*p* = 7.02 × 10^−6^; *Z*-score = 6.16) and 3-oxoacyl-[acyl-carrier-protein] reductase (NADPH) activity, which is important for fatty acid biosynthesis (*p =* 1.13 × 10^−4^; *z* = 8.31) (Table S2). Other functions related to protein synthesis, such as aminoacyl-tRNA editing activity (*p* = 5.38 × 10^−4^; *z* = 6.64), are more represented in LL than HL strains. Aminoacyl-tRNA synthetases (AARS) enzymes synthesize aminoacyl-tRNAs to facilitate translation and maintain translational accuracy through quality control mechanisms, like proofreading. These processes are crucial for cellular viability [29]. The comparative genomic analysis revealed that the gene complement of AARSs and their synthesis routes differed among cyanobacterial species. This diversity was attributed to the presence of duplicated genes, horizontal gene transfers, and gene loss events. For example, AARS genes, such as *GluRS*, *HisRS*, and *ArgRS*, showed evidence of interphylum horizontal gene transfers. These evolutionary adaptations are essential for ensuring accurate and efficient aminoacetylation under different environmental conditions [30], suggesting their crucial role in modifying proteins under low-light conditions. Although a clear explanation is currently not available, one can hypothesize that because protein synthesis is an energy-intensive process and LL strains are under energy-limiting conditions, high copy numbers of AARS can help reduce erroneous protein synthesis. We also observed the enrichment in Pfams for the GO term ‘ATPase activity’ (coupled to the transmembrane movement of ions) (*p* = 3.9 × 10^−3^; *z* = 4.64) (Table S2), which helps maintain ion homeostasis and optimize photosynthesis in deep waters [31]. In the HL strains, we saw unique GO terms enriched in molecular functions related to the different metabolic pathways involved in the response to HL stress. The stress from changes in light intensity alters gene expression patterns, including the genes for RNA metabolism and processing (such as RNA helicases).

DNA repair was one of the top GO terms in the biological processes in HL strains (*p* = 7.78 × 10^−3^; *z* = 2.86); this repair mechanism is essential for this cyanobacterium to recover from ultraviolet (UV) damage and maintain genome integrity [32]. Due to exposure to high UV radiation levels from sunlight, DNA damage is a highly challenging factor for this genus, especially the HL strains [33,34]. Cyanobacteria possess large numbers of genes homologous to *E. coli* DNA recombination and repair genes, such as *uvrABCD*, *recA*, and *recG*, known to form part of their set of core genes [32,35]. One of the primary responses to UV stress is postponing cell division until the completion of the DNA repair by the SOS system, which is a common stress-response pathway also found in *E. coli* [33]. Another enriched term is the nitrogen cycle metabolic process (*p* = 5.64 × 10^−6^; *z* = 8.32) (Table S2), which is important for nitrogen cycling, which transforms nitrogen compounds into amino acids and nucleotides, and is a mechanism essential for growth and survival. Some of the nitrogen-uptake pathways are encoded by flexible genes found in some but not all *Prochlorococcus*. A few HLII strains had nitrate-assimilation genes (in addition to the strains from the LLI clade). The frequency of cells capable of assimilating nitrate is positively correlated with decreased nitrogen availability, which is a limiting factor in surface waters within the HL strains [36]. In the LL strains, we observed enrichment in the GO terms related to metabolic processes and compound biosynthesis and translation elongation, essential for optimizing protein synthesis under low-light conditions. The enrichment of these high-level terms indicates that the ability of *P. marinus* strains to acclimate to different habitats (light intensity and nutrient concentration) has, on a much longer timescale, driven the differentiation of these ecotypes.

### 3.2. Identification of Minimal Pfam Sets Distinguishing HL and LL Strains

**Figure 2:**
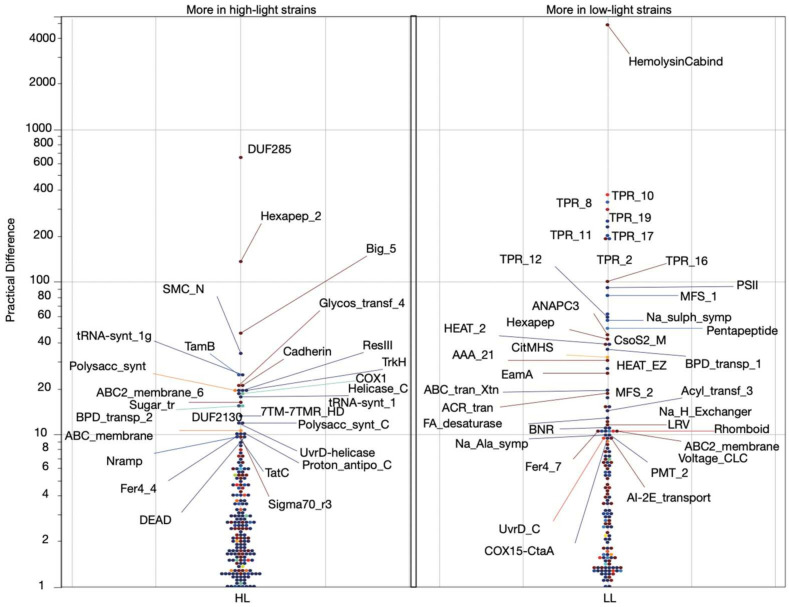
The protein families that significantly differ between the HL and LL groups in FDR- and outlier-corrected batch *t*-tests. The data points are labeled with their Pfam symbol (https://www.ebi.ac.uk/interpro, accessed on 1 October 2023). For example, the LL cluster of points with large practical differences from the HL group contains various tetratricopeptide repeat domains (TPR). These Pfams are consistent with a more heterotrophic lifestyle [37]. The left panel shows Pfams with significantly higher abundances in HL strains, and the right panel shows Pfams with a higher abundances in LL strains.

To delineate the essential Pfam features that differentiate HL- and LL-adapted strains, we performed false-discovery rate (FDR) and outlier-corrected batch *t*-tests using the bit scores from Pfam calls in the *P. marinus* strains (*n* = 40). We aimed to identify a minimal Pfam set that would serve as distinguishing characteristics for HL and LL adaptation. We compared the HL (*n* = 25) and LL (*n* = 15) *Prochlorococcus* strains to determine the main genetic differences indicating adaptation to HL and LL conditions (Figure 2). We determined the most significantly differing Pfams in the two groups (FDR *p* < 0.05; Table S3) to provide a training set for an artificial neural network (ANN) model. Considering the CO_2_ gradient that also differentiates the HL and LL strains, we expected some of the highest variance Pfams to function in the carbon concentration mechanism (CCM). However, none of these Pfams had direct roles in carbon sequestration, indicating that the CCM in the HL and LL strains was generally more conserved than other processes.

**Figure 3:**
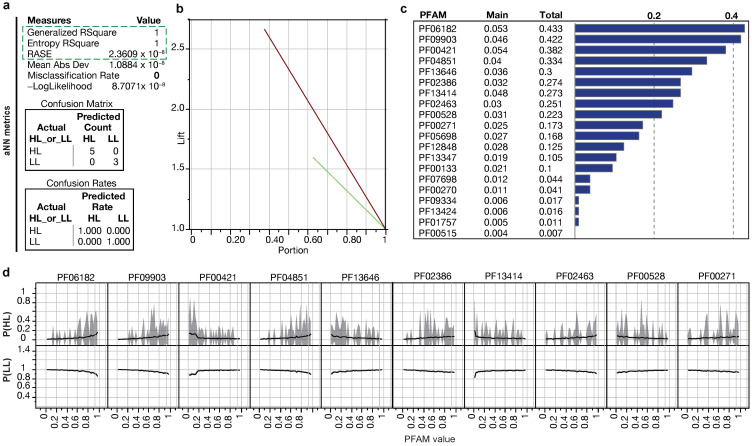
An artificial neural network (ANN) reconstructed from the top ten differing Pfams in HL and LL strains (*n* = 40). (**a**) Performance of the training model using three TanH nodes in a single-layer neural network using a squared penalty and transformed covariates. The input dataset consisted of normalized sum bit scores of Pfams. These metrics describe the performance of the model using a randomly selected 20% holdout set of genomes that were removed before model creation. The model demonstrates a misclassification rate of zero (green box) based on the unknown set of *Prochlorococcus* genomes; (**b**) the lift curves for the training (green) and validation (red) (holdout) sets. The lift curves show how well the variables perform at prediction per sample size; (**c**) assessment of variable importance in the creation of the ANN model. The influence is shown regarding the total effect and main effect for classification; (**d**) marginal model plots for the variables used to create the model. The Y-axes represent probabilities for predicting whether a strain is an HL or LL strain as a function of each Pfam. As the copy number of a Pfam increases, the probability of either HL or LL is determined.

We carried out the supervised training of an ANN model to distinguish between HL and LL groups based on the defined Pfam set. The Pfams were selected from the batch *t*-tests as the top 20 significantly different domains (Table S4). This approach aimed to establish statistical integrity for batch comparisons while generating conclusions distinguishing LL and HL strains with ANNs. Figure 3 displays the top 10 Pfams (of the selected 20) that contribute the most to the ANN decision-making algorithm. These Pfams can discriminate the *P. marinus* genomes based on their depth adaptation, with six Pfams indicating a high likelihood (>99%) of a strain residing in HL conditions and four Pfams suggesting residence in LL conditions (Figure 3). Most of these protein domains have established or predicted roles in photosynthesis.

The Pfam PF06182, an ABC-2 transporter (*p* = 1.34 × 10^−35^), was one of the most representative Pfams in the ANN analysis for being highly prevalent in HL strains (Table S4). The ANN shows that the levels of ABC-2 transporters in *Prochlorococcus* genomes are highly predictive of an HL environment. The ABC-type transporters are upregulated under stress conditions (such as low temperature), comprising a defense system against accumulated toxic metabolites [37]. Considering the other HL-predictive proteins found to be highly influential in distinguishing LL from HL in the ANN model, a reasonable explanation for the higher levels of ABC transporters in HL strains is that at least one of their functions is to remove and replace damaged proteins, aiding in the maintenance and recovery of the optimal photosynthetic efficiency under HL stress.

Among the most significant Pfams for the ANN were PF00271 and PF00270, representing the helicase conserved C-terminal domain and DEAD box helicase, respectively. The helicases use ATP to bind and remodel nucleoprotein–DNA or nucleoprotein–RNA complexes [38,39]. DNA and RNA helicases unwind the double-stranded DNA/RNA regions and are involved in DNA replication, recombination, repair, and overall genome stability [38], and the RNA-dependent helicases participate in various aspects of RNA metabolism [39]. In cyanobacteria, the CrhR-like RNA helicases coordinate the expression of genes required to maintain oxygenic photosynthesis, and the C-terminal domain of CyanoP is involved in carotenoid binding and is a paralog of the C-terminal domain of helical carotenoid proteins (HCPs), which are involved in energy transfer and photoprotection [40]. DEAD box domains are found in cold shock proteins, transcription repair, DNA ligases, and DNA helicases in HL strains.

The Pfams PF00133 and PF09334, annotated as AARS, have increased copy numbers in HL strains (*p* = 1.73 × 10^−17^ and 1.25 × 10^−16^) (Table S4). These Pfams play central roles in cell physiology and may have influenced the origin and evolution of the genetic code. In MED4, the tRNA domains are found in different tRNA ligases (methionine, leucine, isoleucine, valine, and cysteine). AARS genes show evidence of horizontal gene transfer (HGT); in addition, it has been shown that the set of AARS-encoding genes varies from one cyanobacterium to another due to gene gain, loss, or duplication [30]. The higher copy numbers of some of the Pfam-encoding proteins involved in RNA processing and DNA repair in the ANN indicated that a strain was HL. Our results suggest that the HL conditions endured by upper photic cyanobacteria require more copies of the helicase and tRNA synthetases. This can be associated with the regulation of genes involved in stress responses, including the responses to light and nutrient conditions in HL zones.

The most represented Pfams in the LL strains are TPR repeats (PF13414, PF00515, and PF13424 representing TPR_11, TPR_1, and TPR_12 (*p =* 0.000821054; 0.003134574; 0.00053802) (Table S4). The tetratricopeptide repeats’ (TPR) structural motif is present in a wide variety of proteins in procaryotes and eukaryotes [41]. The repeat proteins are known for their extended modular nature, allowing for interactions with different ligands to facilitate the formation of functional complexes [42]. In cyanobacteria, TPR repeats play an important role in PSII assembly and repair [43]. The difference in these TPR repeats between LL and HL strains suggests there are different adaptations used by PSII under different light conditions. In *Synechocystis*, slr0151 (TPR protein) mutants showed reduced photoautotrophic growth and oxygen-production rates under HL stress conditions [43]. We hypothesize that due to their wide variety and ability to bind to diverse ligands, the TPRs specific to LL strains may regulate the assembly and stability of PSII under low-light conditions [44]. Interestingly, a TPR-family membrane protein gene is required for the light-activated heterotrophic growth of the cyanobacterium *Synechocystis* sp. PCC 6803 [45]. Thus, the many proteins we observed with TPR repeats, especially in LL strains, may be important for *P. marinus* heterotrophy and adaptation to the lower oceanic photic zone.

In addition, we observed relatively high levels of the HEAT (Huntingtin, elongation factor 3, protein phosphatase 2A, and TOR1) domain (PF13646) in the LL group (*p* = 7.22 × 10^−22^). HEAT repeats are protein motifs found in a wide spectrum of eukaryotic and prokaryotic proteins and are involved in many cellular processes. While their functions are diverse, a common function of HEAT repeats is thought to be the mediation of protein–protein interactions [46], which are crucial for the assembly of multiprotein complexes. They also play a role in intracellular transport, which differs in the mechanism and regulation between LL and HL strains [47]. While HL strains have evolved mechanisms to maximize energy conversion under HL conditions [48], LL strains have developed strategies to conserve energy in LL conditions [49].

The third most influential Pfam was PF00421, representing the intrinsic antenna proteins CP43 (PsbC) and CP47 (PsbB), which are found in the reaction center of PSII. The higher copy numbers of this Pfam indicated an LL lifestyle (*p* = 1.26 × 10^−61^) (Table S4). PsbC and PsbB antennas transport the excitation energy to the core of the PSII. Some cyanobacteria adapt to LL environments by altering their photosynthetic machinery to absorb far-red light (FRL, 700–800 nm). This process (the far-red-light-acclimation process, FaRLiP) requires the activation of the PsbA, PsbB, PsbC, and PsbD subunits described in *Synechococcus* [50]. Though the PsbC and PsbB proteins do not play a direct role in LL acclimation in *P. marinus*, the observation that PSII is highly predictive of LL status in the ANN suggests a decisive role in *Prochlorococcus*’ adaptation to LL conditions.

The top 20 Pfam contributors to the distinction between HL and LL strains correspond to 43 proteins in MED4 and 64 proteins in NATL2A. The interactome data for these proteins were extracted from the STRING database, revealing distinct protein–protein interaction (PPI) networks for each strain. The MED4 network comprises 38 nodes and 34 edges; meanwhile, the NATL2A network consists of 51 nodes and 94 edges, indicating a higher average clustering coefficient for NATL2A (0.709 compared to 0.669 for MED4). In the NATL2A LL-strain network, a cluster of Pcb proteins is observed, known for their crucial role in light harvesting and adaptation in LL strains. Conversely, the MED4 network displays a smaller cluster of three Pcb proteins compared to seven in NATL2A. Additionally, MED4 features a cluster of UvrA, UvrB, recG, and RecN proteins. The Uvr proteins are part of the highly conserved UvrABC nucleotide excision repair (NER) pathway; meanwhile, RecN and RecG are involved in DNA repair and recombination. These networks illustrate the interactions based on both physical interactions and functional associations. Extracted PPIs, based on co-expression data for both strains, showed seven PPIs for NATL2A and twelve PPIs for MED4. These interactions are derived from orthologous protein interactions in various organisms, such as *Synechococcus*, *Arabidopsis thaliana*, and *B. subtilis*. Subnetworks of tRNA synthetases are found in both strains, with ileS and leuS present in both subnetworks, indicating their essential roles across both strains (Figure S1). valS is unique to NATL2A, and its interaction with ileS and leuS suggests a tightly regulated network of AARS specific to the LL strain, possibly reflecting the adaptations to optimize protein synthesis under LL conditions. In the HL strain, the presence of metS (unique to MED4) together with ileS and leuS suggests a different regulatory mechanism that emphasizes efficient translation initiation and potentially faster growth or protein synthesis under HL conditions.

This inclusion of interspecies data suggests that while the core proteins might be conserved, their interaction partners and functional dynamics can vary significantly between HL- and LL-adapted strains, potentially leading to different physiological adaptations and responses to environmental light conditions.

### 3.3. Variable, Depth-Dependent Accumulation of Endogenous Viral Elements

**Figure 4:**
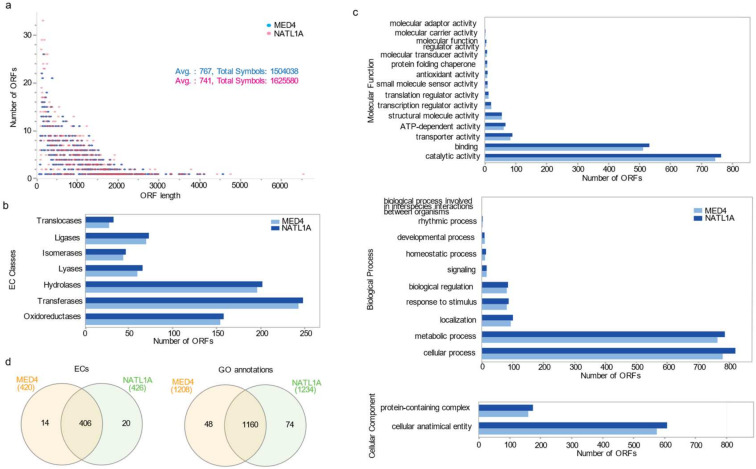
The sequence length distribution and EC class GO term distribution among the MED4 and NATL1A ORFs. Panel (**a**) represents the sequence length distribution with an average of 767 bp and 741 bp for MED4 and NATL1A, respectively. Classification of the enzyme commission numbers assigned to the ORF sequences to MED4 and NATL1A; (**b**) representation of GO annotation level distribution after GO-Slim for MED4 and NATL1A; (**c**) the number of sequences (X-axis) and the GO terms (Y-axis) for biological processes, molecular functions, and cellular compartment ontologies are shown for both strains; (**d**) functional annotation analysis using Blast2GO. Comparison between the MED4 and NATL1A strains based on GO annotations and enzyme commission (EC).

Cyanobacterial viruses [51] exhibit considerable effects on the evolution, lifecycle [52], and ecosystem dynamics [53] of widespread cyanobacterial populations. Viral sequences can integrate into their hosts’ genomes [54]; thus, we examined the *P. marinus* genomes for viral family sequence differences in strains (Figure 4). Their abundances were compared with response screening. In the *P. marinus* genomes, we observed many viral families (VFams) from viruses infecting eukaryotic hosts [10] (Table S4). Although these eukaryotic viruses were not expected to infect prokaryotes, the exchange of genetic material may have still occurred in the ancestors of *P. marinus*, especially with giant viruses [54,55,56]. The genomes of giant DNA viruses contain up to 1.5 Mbp of DNA. In the open ocean, they constitute large populations that, through lysis, release enormous amounts of DNA, which can then be taken up by cyanobacteria.

*P. marinus* genomes showed several highly conserved VFams, including VFams 5317 (methionyl-tRNA synthetase; *Megavirus lba* [57] and *Acanthamoeba polyphaga mimivirus* [58]), 5363 (phospho-2-dehydro-3-deoxyheptonate aldolase; *Pandoraviruses*), 4000 (adenosylhomocysteinase; *Pandoraviruses* [59]), 126 (HSP70, conserved in plant viruses, including *Ampelovirus*, *Closterovirus*, and *Crinivirus*), 2918 (AAA family ATPase; *Acanthamoeba polyphaga moumouvirus* and *Megavirus chiliensis*), 183 (DNA topoisomerase II; *Marseillevirus* [60], *Wiseana iridescent virus, Lausannevirus*), 917 (glutamine:fructose-6-phosphate amidotransferase; *Paramecium bursaria Chlorella virus 1*, *Phaeocystis globosa virus*, *Megavirus lba*), and 3355 (fucose synthetase; *Paramecium bursaria Chlorella virus 1* [61]). These conserved VFams in *P. marinus* are probably necessary for the survival of the species worldwide.

Two clusters of VFams were unique to LL strains, including strains 760155.1LL II/III (LG), 760255.1LL II/III (SS2), 760355.1LL II/III (SS51), and 11485.1LL IV (MIT9313). These unique VFams included VFam_5531 (Rad5-like protein; *Pandoraviruses*), VFam_1166 (2OG-Fe(II) oxygenase family protein; *Phaeocystis globosa* virus [62], *Ostreococcus* viruses [63]), VFam_236 (ATPase; *Ectocarpus siliculosus* virus 1 [64]), VFam_3032 (putative DNA helicase, *Megaviruses*), VFam_517 (NAD-dependent DNA ligase; *Betaentomopoxviruses* [65], *Iridoviruses*), VFam_5367 (guanine deaminase; *Pandoraviruses*), and VFam_3949 (SAM-dependent methyltransferase; *Megaviruses*). The selective uptake or retention of genes with these VFams in the LL species suggests they facilitate cyanobacterial survival at depth.

We extracted all ORFs from the 40 samples of the *P. marinus* genomes containing VFams. These ORFs were examined for the Pfams used in the ANN model. We observed a mean of 15.3 VFams/genome on ORFs that also had ANN Pfams. The Pfams with VFam codomains mainly consisted of ORFs annotated as methionine tRNA ligases, ATP-dependent and DEAD/DEAH-box helicases, and cold-shock proteins. These Pfams significantly varied among the HL and LL groups and were included in the ANN set. Interestingly, the LL strains had an average of 11.8 ANN-linked VFams more than the HL strains (*t* ratio = 2.4 and *p* = 0.0319 in a two-tailed *t*-test). The significantly higher level of VFams from these genes suggests their involvement in distinguishing HL and LL groups. The putative viral (phage) origin for their codomain Pfams outlines a scenario where phage-acquired genes were instrumental in the adaption of the HL and LL *Prochlorococcus* strains to their respective niches.

### 3.4. The Prochlorococcus Strains MED4 and NATL1A as HL and LL Representatives

To explore the differences between the HL and LL *P. marinus*, we invested in analysis of the genomes of MED4 (HL-adapted strain) and NATL1A (LL-adapted strain). We extracted functional annotation using Blast2GO (https://www.blast2go.com/, accessed on 25 November 2022) [17], with the complete gene sequences available from EnsemblBacteria (https://bacteria.ensembl.org/, accessed on 1 February 2019). The average length distribution for genes is 767 bp and 741 bp for MED4 and NATL1A, respectively (Figure 4a and Table S6), with 92% of the cDNA sequences smaller than 1.5 kb (1809/1960 of MED4 and 2024/2193 of NATL1A cDNA sequences). The B2GO analysis annotated 1514 and 1593 sequences of MED4 and NATL1A, respectively, with 1123/1177 (MED4/NATL1A) assigned gene ontology terms (GO terms) and 869 and 902 enzyme commissions (ECs). The latter values correspond to 724 and 751 sequences among the available annotated genes for MED4 and NATL1A, respectively (Table S6). A total of 446 and 600 sequences could not be annotated in MED4 and NATL1A, respectively. The EC distribution analyses showed similar distributions between both strains for different enzymatic classes and sub-classes (Figure 4b). We analyzed the EC overlap using Interactive Pathway Explorer version 3 (iPath3) (Figure S2), and similar to the enrichment illustrated in the Venn diagram in Figure S3, the EC map mostly identified common metabolic pathways. The unique metabolic pathways differed in either specific modules or enzymatic reactions. One example is the amino sugar and nucleotide metabolism pathway that forms part of the carbohydrate pathway shown to be unique in some modules in MED4 and NATL1A (highlighted in blue and red in the metabolic map in Figure S2). Though a purine metabolism pathway is common to both the HL and LL strains (highlighted in green), UDP-N-acetyl-D-glucosamine 2-epimerase is unique to NATL1A, while UTP:N-acetyl-alpha-D-glucosamine-1-phosphate uridylyltransferase is specific to MED4.

We found that N-glycan biosynthesis (highlighted in blue in the glycan synthesis and metabolism in the metabolic map; Figure S2) was unique to the HL strains with no overlap with the LL strain. Glycans are complex carbohydrates with high structural diversity; methylation, acetylation, or the addition of sulfate groups enhance their diversity. Their presence at the cell surface confers adaptability to a variety of environmental factors [66]. The glycans in the extracellular polysaccharide layer can play a protective role against desiccation, guarding against ROS under UV-A or UV-B irradiation [28]. Another example showing the unique modules and reactions to the NATL1A strain is highlighted in red within the area of carbohydrate metabolism shown in the metabolic map, Figure S2, within the glycolysis, pyruvate metabolism, and carbon fixation pathways.

Gene ontology (GO) hierarchies for HL and LL strains showed mostly similar GO distributions for MED4 and NATL1A represented by three GO terms for biological processes (BPs), molecular functions (MFs), and cellular components (CCs) (see Figure 4c and Table S6). The GO levels provide a broad overview of the ontology content from the minimal GO-Slim ontology set. We found similar GO-term distributions for MED4 and NATL1A among the three different categories (BP, MF, and CC), with a slightly higher number of sequences representing the top two GO terms in each category for the NATL1A strain (Figure 4c and Table S6). We compared the number of encoded annotated enzymes for both strains and found that only 14 and 20 ECs are unique to MED4 and NATL1A, respectively. In comparison, most ECs are shared between the two genomes (406 ECs) (Figure S3). GO terms had similar distributions in both MED4 and NATL1A. The majority of GO terms were shared among the strains, while only 48 and 74 were unique for MED4 and NATL1A, respectively (Figure S3). The common GO terms were related to essential survival functions, while species-specific GO sets were related to processes involved in the adaptation of these strains to their local environmental conditions.

### 3.5. ORFeome Resource Development

To complement our computational analyses, we synthesized the complete ORFeomes for the *P. marinus* strains MED4 and NATL1A to serve as biological resources for the downstream studies. The *Prochlorococcus* species have the most compact genomes of free-living, oxygenic phototrophs. MED4, an HL-adapted strain, is the smallest with 1.65 Mbp and 30.8 G + C%, whereas NATL1A is an LL-adapted strain with a slightly larger genome of 1.86 Mb and a higher GC content of 35%. The genomes of MED4 and NATL1A consist of a single circular chromosome and encode ~1790 and ~1976 genes, respectively [13]. To synthesize the MED4 and NATL1A ORFs, we extracted structural gene annotation and cDNA sequences for complete genomes from the EnsemblBacteria database (https://bacteria.ensembl.org/, accessed on 1 February 2019, Cambridge, UK) accessed on 1 February 2019), resulting in a list of 1960 and 2193 genes for MED4 and NATL1A, respectively. The open reading frame sequences ranged from 80 to 4500 bp with an average length of 760 bp for MED4 and from 100 bp to 6 kb with an average length of 1260 pb for NATL1A. The ORFs were flanked with ATTL1 and ATTL2, allowing recombinational sub-cloning into a variety of Gateway^®^-compatible vectors [67]. The ORFs were synthesized in the pTWIST-ENTR vector (twistbioscience.com, San Francisco, CA, USA), providing a Gateway^®^ cloning compatible system [68]. The stop codons were removed from the cDNA sequence, and codon fitting was applied for optimal synthesis (Figure S4). Approximately 99% of the attempted ORFs were successfully synthesized and sequence-verified, with 1826 out of 1840 ORFs and 2150 out of 2193 ORFs for MED4 and NATL1A, respectively.

To validate the recombinational transferability of ORFs, a set of 70 ORFs of the MED4 strain was recombinationally cloned into two different yeast two-hybrid Gateway^®^-compatible vectors, pAD and pDB (Figures S4 and S5) [69]. We randomly selected half of the re-cloned ORFs and their respective parental ORFs, using Sanger sequencing with both forward and reverse vector-specific primers, and observed 90% alignment to the reference ORF sequences.

MED4 and NATL1A ORFeomes are available as bacterial glycerol stocks; their respective distribution and plate maps are illustrated in Tables S7A and S7B. Users can find further information about each ORF, their plate reference (well and plate number), their corresponding sequence, gene reference, and related information from the National Center for Biotechnology Information (NCBI).

## 4. Discussion

**Figure 5:**
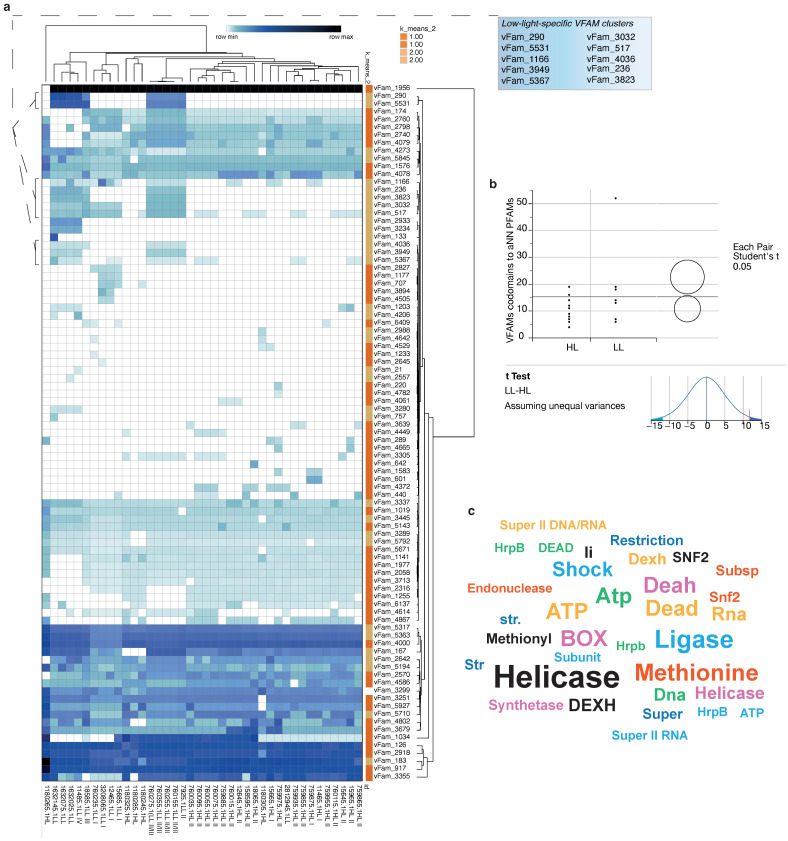
The viral families (VFams) in *P. marinus* strains (*n* = 40), including NATL1A and MED4. Most VFams were highly conserved in all *P. marinus* strains except for two distinct clusters unique to some LL strains. (**a**) Hierarchical bi-clustering of HMM match bit scores. The LL group had unique clusters of VFams (shown in the adjacent box); (**b**) VFams from genes in the ANN Pfam set (Figure 4). Circles represent group distributions and means in statistical comparisons. The overlap between circles indicates whether the means are significantly different (non-overlapping) or not significantly different (overlapping), providing an intuitive representation of pairwise comparison results. We identified that LL strains had significantly more VFams related to these influential Pfams; (**c**) word cloud generated from the annotations of genes containing both VFams and ANN Pfams. Several types of helicases were over-represented in this gene set.

The shifts in phytoplankton communities serve as indicators of climate-induced disruptions in the marine ecosystems [1]. Cyanobacteria are highly sensitive to environmental changes, including light, temperature, and nutrient availability. Studying their adaptation to different environmental conditions helps predict shifts in phytoplankton distributions in marine ecosystems [70]. Despite cyanobacteria being a model for studying essential cellular mechanisms, including photosynthesis, plant evolution, and carbon fixation, the adaptive evolution of the species with respect to depth is not adequately understood [20,71,72,73]. Cyanobacteria often have multiple, seemingly redundant copies of genes in their genomes; additionally, these genes are rarely paralogs. Instead, they are more likely to be independent acquisitions from the ubiquitous clouds of cyanophages that surround cyanobacteria in their native environment [74]. For this reason, focusing on Pfams instead of segments of genomic sequences can bring new insights that would not be detected using paralog analyses [75]. To identify the genomic determinants of the cyanobacterial adaptation to LL and HL conditions, we used *Prochlorococcus marinus* strains as models and explored their 40 sequenced and published genomes. We analyzed the domains and functional regions and uncovered differences in the genomic signatures between LL and HL strains. In addition, we performed Pfam analyses, which provide information on the identity and number of coded protein domains. We augmented these analyses through the reconstruction of ANNs that could accurately distinguish between the HL and LL status using a subset of Pfam predictors, thereby identifying the small number of features that are sufficient to distinguish HL and LL strains. The most influential Pfams were quantified, and the results show that the Pfams with the highest predictive capacity were mostly photosynthesis-related (predictive of HL status (pHL)) or involved in redox (i.e., electron transfer) processes (predictive of LL status (pLL)) (Figure 5).

Over the past centuries, human activities relating to urbanization, agriculture, and industrial development have led to an excessive influx of nutrients, especially nitrogen and phosphorus [76], in aquatic systems, along with a dramatic increase in atmospheric levels of CO_2_ [77]. This nutrient over-enrichment has resulted in eutrophication (an acceleration in the rate of primary production), which in turn can lead to an increase in the growth of blooms of harmful cyanobacteria [76]. In addition, due to climate change and ocean warming, stratification is leading to an increase in light availability in the upper levels of the ocean environment and the reduced transport of nutrients [78]. To gain a fuller understanding of how phytoplankton may respond to these changes and to generate biological resources for downstream wet-bench experiments, we expanded the genome analysis for 40 *P. marinus* strains, applied bi-clustering to detect differential gene content in the HL and LL strains of *P. marinus*, and generated genome-wide ORFeome resources for NATL1A and MED4, as representative LL- and HL-adapted species of the *Prochlorococcus* genus. Cyanophage genomes contain abundant accessory metabolic genes (AMGs) presumably used to manipulate their hosts’ metabolism. We hypothesize that cyanophage populations act as genetic reservoirs for *Prochlorococcus* and are partly responsible for the differential genetic contents in HL and LL strains. The results from our analysis of the viral families in *Prochlorococcus* support this hypothesis with a substantial functional overlap with the total differential gene content between the HL and LL groups.

Our analyses showed that most VFams are highly conserved among the strains. We also identified distinct clusters in the LL strains where the enriched VFams highlight their viral origin. Phages play an essential role in the evolution of microbial communities, including cyanobacteria, and cyanophages are among the key factors determining the rate and direction of evolution in cyanobacterial populations [79]. Among the identified viral domains, some isoforms can also be found in HL strains (involved in DNA repair or light-harvesting systems), but they were not detected in the VFam analysis. This finding suggests that the transfer of some protein domains has undergone convergent evolution, explaining their differential origins in LL and HL strains [11]. These studies have shown that marine viruses serve as a potential genetic pool in shaping the evolution of cyanobacterial photosynthesis. Some LL *P. marinus* strains have acquired many of their genes from other cyanobacteria, such as *Synechococcus*, placing these LL strains closer to *Synechococcus* than the HL strains [80]. The phage intermediates are involved in the transfer and shuffling of genes encoding photosynthetic proteins (*psbA*) between *Prochlorococcus* and *Synechococcus* [81]. Other studies have shown the dynamic co-evolutionary process in both host and phages [82], explaining the differences in the evolution and genomic differences among *P. marinus* strains.

We used functional annotation from available genome sequencing to compare MED4 and NATL1A genomes on both a functional and metabolic level, which revealed a high degree of similarity for both strains when they were compared for their respective encoded enzymes and GO-term functions. In addition, metabolic pathway analyses permitted the dissection of the metabolic pathway implications for available EC annotations and confirmed a majority of shared metabolism for MED4 and NATL1A, including energy, amino acid, and fatty acid metabolism (Figure S2). In addition to the role of ECs in representing a comparable distribution among enzymatic classes and sub-classes for the two strains (Figure 4b), while both strains are characterized by unique pathways that differ in specific enzymatic reactions, they also share metabolic pathways, such as glycolysis and galactose metabolism, among the unique reactions (Figure S2).

The computational analyses of the available annotated sequences for *P. marinus* strains provided comparative descriptions of their genes, enzymes, and functions; however, in many cases, these are still lacking experimental validation. ORFeome resources allow for a more in-depth examination of the differences and similarities between the *P. marinus* strains through the exploration of their potential interactions, the wiring of protein interactions in signaling pathways, and their implications in cellular and biological functions. Despite being model organisms for studies on photosynthesis and evolution, comprehensive ORFeome resources have not been made available outside the present work. The availability of complete genome sequences and the reduced cost of DNA synthesis allowed us to successfully generate the complete ORFeomes of MED4 and NATL1A. The ORFs are available as DNA clones in Gateway^®^-compatible vectors (from TWIST Bioscience, San Francisco, USA and the Genome Synthesis and Editing Platform, China National GeneBank (CNGB), BGI-Research, Shenzhen, China), which offers the advantage of sub-cloning the genes into various expression vectors. Therefore, the genes can be used in a wide range of studies and for multiple applications. The generation of cDNA libraries has been completed for various organisms, from *A. thaliana* [83,84] to *Homo sapiens* [15,85]. The efforts to achieve complete ORFeome synthesis have been described in relation to several model organisms. These clones are stored in Gateway^®^-compatible vectors, enabling the high-throughput transfer of ORFs into various expression vectors. The usefulness of ORFeome collections is demonstrated by the generation of successive human ORFeome versions, which have facilitated the biophysical proteome-wide studies of protein–protein interactions and related human cellular mechanisms [85,86]. ORFeome libraries have been generated for other model organisms, such as *E. coli* [87], *Drosophila melanogaster* [88], and *Chlamydomonas reinhardtii* [89]. These efforts were followed by generating databases and interfaces that provide integrated sets of bioinformatic tools to analyze and clone ORFs. For example, the ViralORFeome (https://omictools.com, accessed on 1 October 2023) provides a framework to establish an extensive collection of the ORFs for viruses [90]. The tool provides an interface to define ORFs and design ORF-specific cloning primers, such as the specific one for virus genome sequences. The development of technologies in DNA synthesis methods has progressed from the template-based DNA amplification and cloning approaches to de novo gene synthesis, allowing codon optimization with a higher DNA yield, increased oligonucleotide length (up to 5 kb), and improved success rate [91]. For example, the synthesized metabolic ORFeome of *C. reinhardtii* was generated with a 70% success rate (using reverse-transcription PCR, amplification, and cloning) [89]. In comparison, the de novo synthesis of the MED4 and NATL1A ORFeomes resulted in a 99% success rate. The industrialization of DNA synthesis has also resulted in cost reductions and accelerated workflows, making the synthesis of large-scale genes and ORFeomes accessible and affordable for synthetic biology applications.

These ORFeome libraries can have various experimental applications. The ORFeomes can be used to systematically study gene functions in MED4 and NATL1A. For example, the overexpression or knocking out of specific ORFs contributes to the investigation of the roles of individual genes in the stress response to UV radiation, nutrient limitations, or temperature fluctuations. Another available application is interactome mapping for protein–protein interactions, using high-throughput yeast two-hybrid screens or co-immunoprecipitation assays to map the protein networks. This can reveal how proteins collaborate in the regulation of key biological functions and how these networks differ between HL- and LL-adapted strains. The comparative analysis of the overexpression and function of the MED and NATL1A ORFs would allow for the genetic and proteomic adaptations to different light conditions to be identified.

The newly synthesized ORFeomes for *P. marinus* MED4 and NATL1A are enabling resources to interrogate the biological mechanisms of adaptation to HL or LL, as well as depth-associated environmental variabilities. In this paper, broad comparative genomic analyses were presented; however, it is the synthesized ORFeomes that provide the means for both high-throughput and focused biological/biochemical experiments to explore the functional roles of genes in the representative HL and LL *Prochlorococcus* species.

## Data Availability

Supporting data generated in this work can be found in Supplementary Tables S1 to S7 (A and B) and Supplementary Data S1, S2, and S3.

## Supplementary Materials

The following supporting information can be downloaded at: https://www.mdpi.com/article/10.3390/microorganisms12081720/s1, Figure S1. STRING protein–protein interaction (PPI) networks for MED4 and NATL2A. Figure S2. The metabolic pathway representation for MED4 and NATL1A using iPath3. Figure S3. GO-term enrichment analysis comparing Pfams distinguishing HL and LL *P. marinus* strains. Figure S4. A schematic representation of ORF entry clones and cloning into Y2H expression vectors. Figure S5. The cloning and transformation of a selected set of ORFs for MED4 strains. Table S1. The bit scores for the predicted Pfam domains from the 40 queried strains. Table S2. The GO-term enrichment results. Table S3. The response screening results. Table S4. The ANN top Pfam contributors. Table S5. The VFam scores. Table S6. Blast2GO analyses results. Table S7. (A) MED4 ORFeome. ORFeome library. (B) NATL1A ORFeome library. Data S1. Pfam annotations. Data S2. The response screening JMP project files. Data S3. The Python scripts.
