# Supplementary material for: Adaptive Evolution Signatures in Prochlorococcus: Open Reading Frame (ORF)eome Resources and Insights from Comparative Genomics
Source: Microorganisms. 2024 Aug 20;12(8):1720. doi: 10.3390/microorganisms12081720 (PMC11357015; doi:10.3390/microorganisms12081720)
Supplement: Supplementary file 1 [file microorganisms-12-01720-s001.zip › Figure S1.pdf]

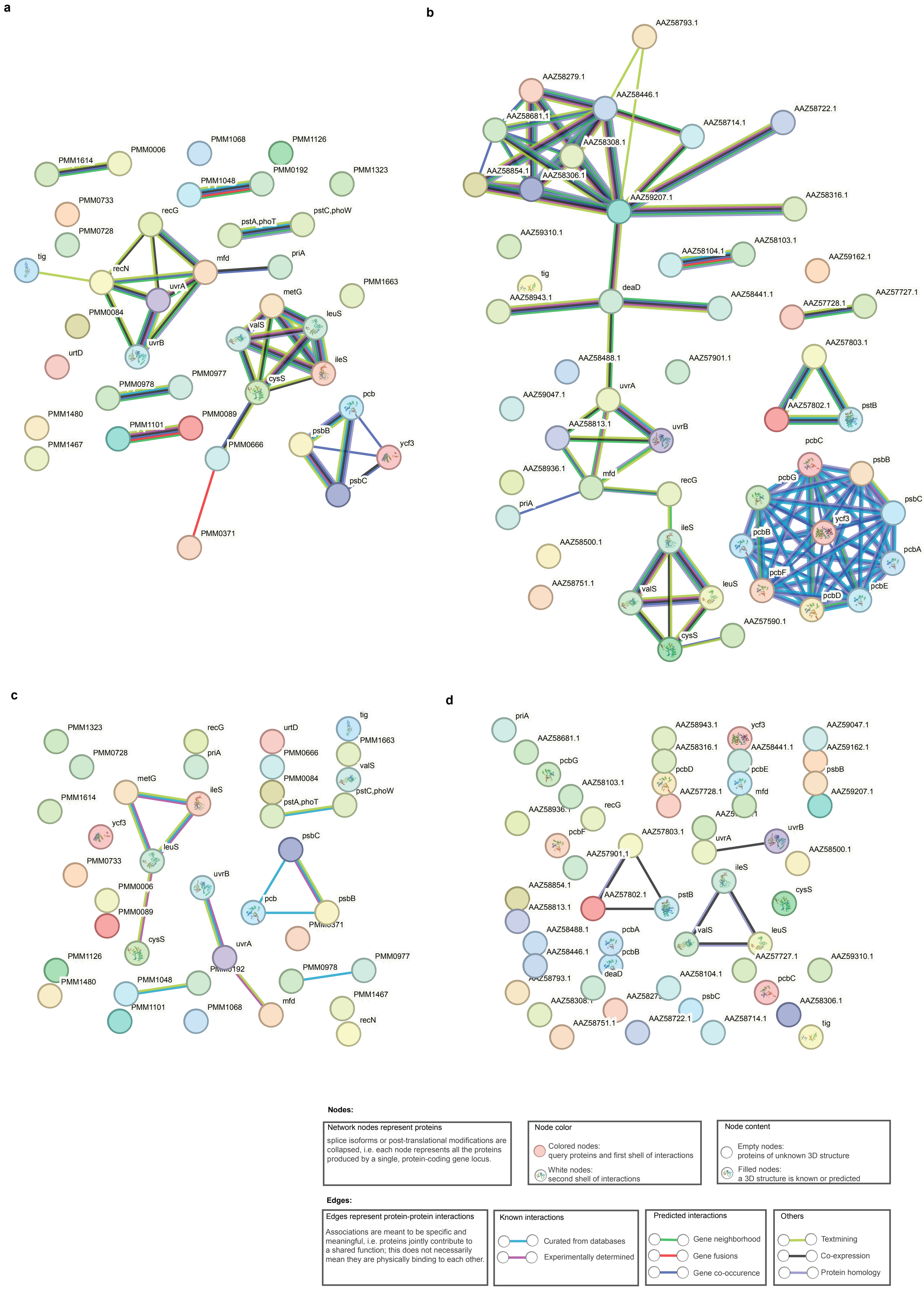
a
PMM1614
PMM0006
PMM1068
PMM1126
PMM1048
PMM0192
PMM1323
PMM0733
recG
pstA,phoT
pstC,phoW
PMM0728
tig
recN
mfd
priA
uvrA
PMM1663
PMM0084
metG
valS
leuS
urtD
uvrB
ileS
PMM0978
PMM0977
cysS
PMM1480
PMM1101
PMM0089
pcb
PMM1467
PMM0666
psbB
ycf3
psbC
PMM0371
b
AAZ58793.1
AAZ58279.1
AAZ58446.1
AAZ58681.1
AAZ58714.1
AAZ58722.1
AAZ58308.1
AAZ58854.1
AAZ58306.1
AAZ59207.1
AAZ58316.1
AAZ59310.1
AAZ58104.1
AAZ58103.1
AAZ59162.1
tig
deaD
AAZ58943.1
AAZ58441.1
AAZ57728.1
AAZ57727.1
AAZ57803.1
AAZ58488.1
AAZ57901.1
uvrA
AAZ59047.1
AAZ57802.1
pstB
AAZ58813.1
uvrB
pcbC
psbB
pcbG
AAZ58936.1
mfd
recG
psbC
priA
ycf3
ileS
pcbB
AAZ58500.1
pcbA
pcbF
AAZ58751.1
leuS
valS
pcbE
pcbD
AAZ57590.1
cysS
c
PMM1323
recG
urtD
tig
PMM1663
PMM0728
priA
PMM0666
valS
metG
PMM0084
ileS
PMM1614
pstA,phoT
pstC,phoW
ycf3
leuS
psbC
PMM0733
uvrB
PMM0006
pcb
psbB
PMM0089
PMM0371
cysS
uvrA
PMM1126
PMM0978
PMM0977
PMM1480
PMM1048
mfd
PMM1467
PMM1101
PMM1068
recN
d
priA
AAZ58943.1
ycf3
AAZ59047.1
AAZ58681.1
AAZ58316.1
AAZ58441.1
AAZ59162.1
pcbG
pcbD
pcbE
psbB
AAZ58103.1
AAZ57728.1
mfd
AAZ59207.1
recG
AAZ58936.1
uvrB
pcbF
AAZ57803.1
uvrA
AAZ58500.1
AAZ57901.1
ileS
AAZ58854.1
AAZ57802.1
pstB
cysS
AAZ58813.1
AAZ58488.1
pcbA
valS
leuS
AAZ58446.1
pcbB
AAZ57727.1
AAZ59310.1
deaD
AAZ58793.1
AAZ58104.1
pcbC
psbC
AAZ58308.1
AAZ58306.1
AAZ58751.1
AAZ58722.1
AAZ58714.1
tig
Nodes:
Network nodes represent proteins
splice isoforms or post-translational modifications are collapsed, i.e. each node represents all the proteins produced by a single, protein-coding gene locus.
Node color
Colored nodes:
query proteins and first shell of interactions
White nodes:
second shell of interactions
Node content
Empty nodes:
proteins of unknown 3D structure
Filled nodes:
a 3D structure is known or predicted
Edges:
Edges represent protein-protein interactions
Associations are meant to be specific and meaningful, i.e. proteins jointly contribute to a shared function; this does not necessarily mean they are physically binding to each other.
Known interactions
Curated from databases
Experimentally determined
Predicted interactions
Gene neighborhood
Gene fusions
Gene co-occurence
Others
Textmining
Co-expression
Protein homology
