# Supplementary material for: Adaptive Evolution Signatures in Prochlorococcus: Open Reading Frame (ORF)eome Resources and Insights from Comparative Genomics
Source: Microorganisms. 2024 Aug 20;12(8):1720. doi: 10.3390/microorganisms12081720 (PMC11357015; doi:10.3390/microorganisms12081720)
Supplement: Supplementary file 1 [file microorganisms-12-01720-s001.zip › Figure S5.pdf]

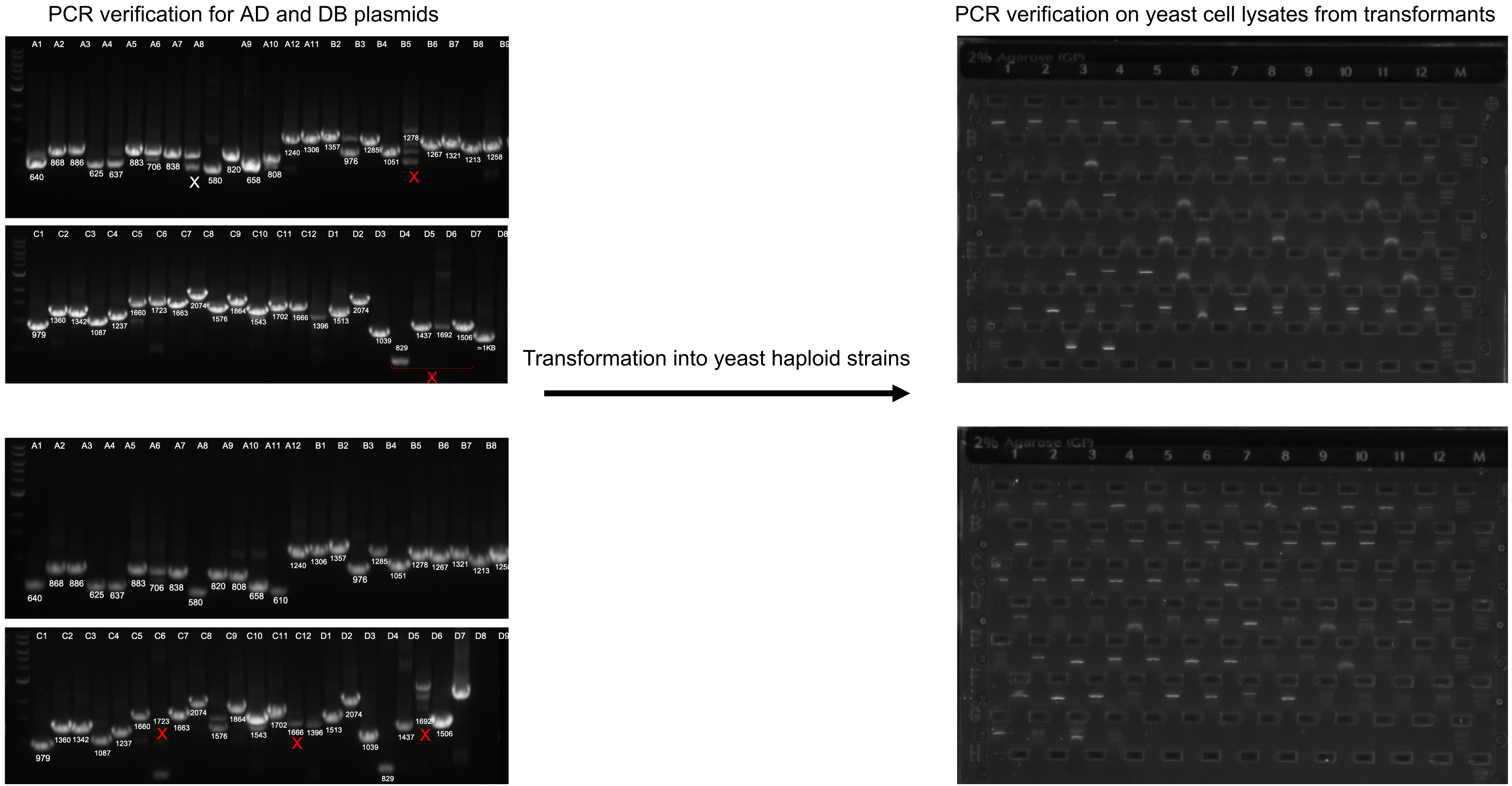

PCR verification for AD and DB plasmids
Transformation into yeast haploid strains
PCR verification on yeast cell lysates from transformants
2% Agarose (GP)
