# Supplementary figures and images for: Adaptive Evolution Signatures in Prochlorococcus: Open Reading Frame (ORF)eome Resources and Insights from Comparative Genomics
Source: Microorganisms. 2024 Aug 20;12(8):1720. doi: 10.3390/microorganisms12081720 (PMC11357015; doi:10.3390/microorganisms12081720)

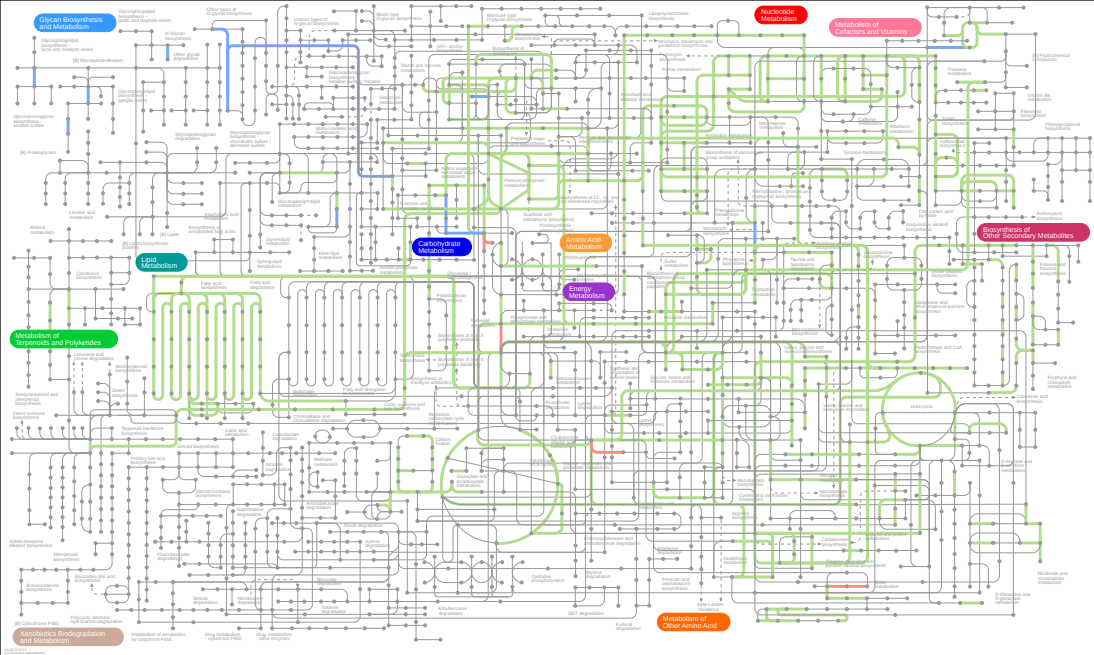

Supplement: Supplementary file 1 [file microorganisms-12-01720-s001.zip › Figure S2.pdf]

**GO terms**  
**Biological Process**

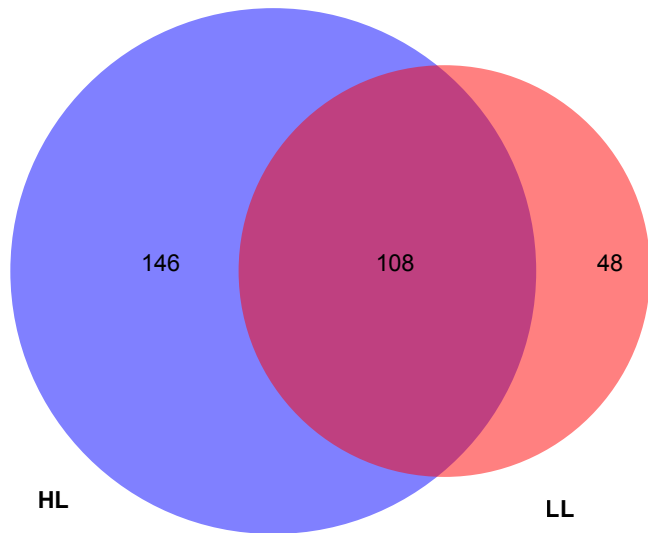

**GO terms**  
**Molecular function**

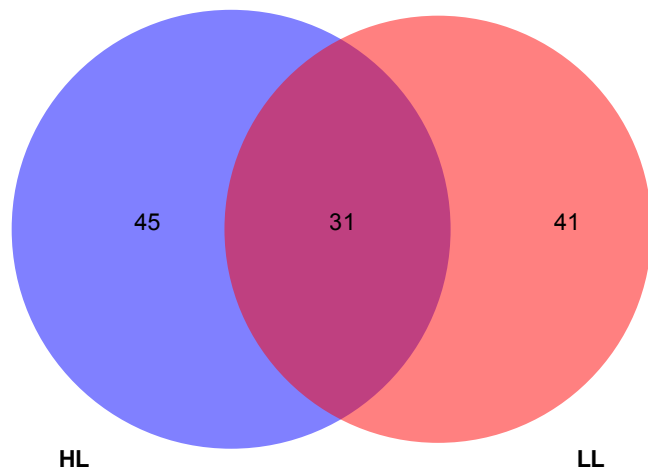

Supplement: Supplementary file 1 [file microorganisms-12-01720-s001.zip › Figure S3.pdf]

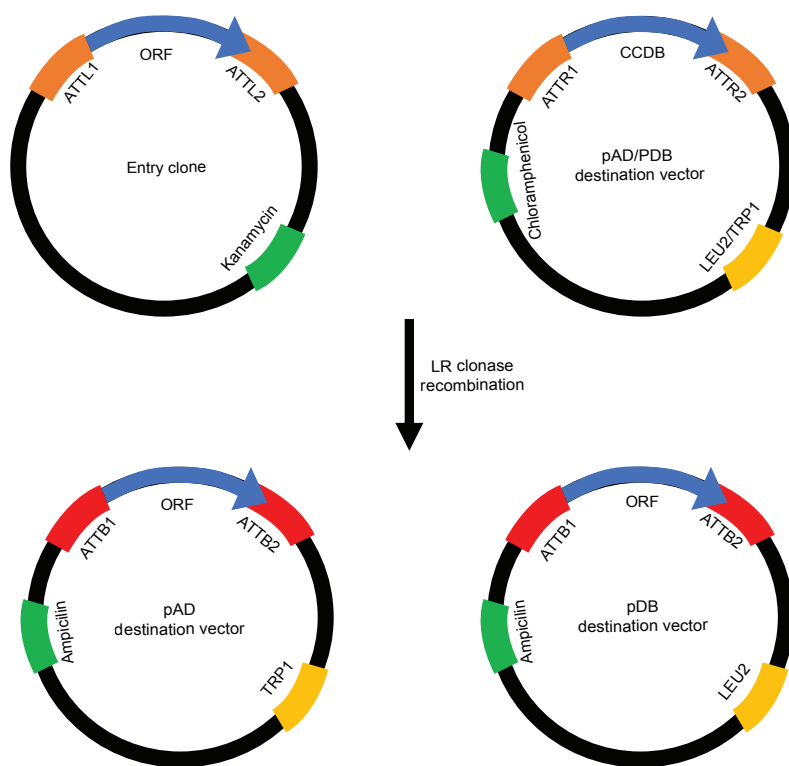

Supplement: Supplementary file 1 [file microorganisms-12-01720-s001.zip › Figure S4.pdf]
